# Transcriptome analysis of tea (*Camellia sinensis*) leaves in response to ammonium starvation and recovery

**DOI:** 10.3389/fpls.2022.963269

**Published:** 2022-08-30

**Authors:** Yu Wang, Jia-Xue Ouyang, Dong-Mei Fan, Shu-Mao Wang, Yi-Min Xuan, Xiao-Chang Wang, Xin-Qiang Zheng

**Affiliations:** ^1^College of Agriculture and Biotechnology, Tea Research Institute, Zhejiang University, Hangzhou, China; ^2^Institute of Dafo Longjing, Xinchang, China

**Keywords:** transcriptome, ammonium, tea plants, RNA-sequencing, nitrogen, WGCNA

## Abstract

The tea plant is a kind of ammonium-preferring crop, but the mechanism whereby ammonium (NH_4_^+^) regulate its growth is not well understood. The current study focused on the effects of NH_4_^+^ on tea plants. Transcriptomic analysis was performed to investigate the early- and late-stage NH_4_^+^ deprivation and resupply in tea plants shoots. Through short- and long-term NH_4_^+^ deficiency, the dynamic response to NH_4_^+^ stress was investigated. The most significant effects of NH_4_^+^ deficiency were found to be on photosynthesis and gene ontology (GO) enrichment varied with the length of NH_4_^+^ deprivation. Enriched KEGG pathways were also different when NH_4_^+^ was resupplied at different concentrations which may indicate reasons for tolerance of high NH_4_^+^ concentration. Using weighted gene co-expression network analysis (WGCNA), modules related to significant tea components, tea polyphenols and free amino acids, were identified. Hence, NH_4_^+^ could be regarded as a signaling molecule with the response of catechins shown to be higher than that of amino acids. The current work represents a comprehensive transcriptomic analysis of plant responses to NH_4_^+^ and reveals many potential genes regulated by NH_4_^+^ in tea plants. Such findings may lead to improvements in nitrogen efficiency of tea plants.

## Introduction

Nitrogen is an indispensable nutrient element for plant growth and represents a major driving force for crop yield improvement (Li et al., 2017). Its absorption and assimilation are similarly important for growth of tea plants. In addition to effects on plant growth and development, nitrogen nutrition also affects their ability to cope with environmental challenges (Vega et al., 2015). Nitrogen affects growth of plant roots and leaves (von Wiren et al., 2000; Menz et al., 2016; Coleto et al., 2017), senescence rate (Vanacker et al., 2006) and flowering time (Castro Marin et al., 2011). The two main sources of inorganic nitrogen for plants are nitrate (NO_3_^–^) and ammonium (NH_4_^+^) and different plants have different preferences for them. For example, maize and tomato grow better with nitrate, while rice prefers NH_4_^+^ (Britto and Kronzucker, 2002). Previous studies have demonstrated the signaling role of NH_4_^+^ (Ren et al., 2020), which induces a variety of morphological and physiological responses (Liu and von Wirén, 2017). For example, NH_4_^+^ inhibited root growth and affected cell elongation and division in primary roots (Li et al., 2010) but local NH_4_^+^ supply increased lateral root initiation and higher-order lateral root branching (Lima et al., 2010). NH_4_^+^ also promoted the alleviate of toxicity by hormones, such as auxin (Esteban et al., 2016).

Tea is a favored beverage throughout the world. The harvested leaves contain many primary and secondary metabolites, such as catechins, theanine and caffeine. A great deal of research has investigated regulatory mechanisms behind the biosynthesis of metabolites, including the regulation of nutrient content (Ruan et al., 2010). Tea plants have been reported to be well adapted to high NH_4_^+^ concentrations and grow better with a supply of NH_4_^+^ (Fan et al., 2015), a characteristic confirmed by the ^15^N study of hydroponic tea plants (Tang et al., 2020). Previous studies found a better nitrogen performance of NH_4_^+^ in tea plants, with increased free amino acids content and reduced secondary metabolites content (Ruan et al., 2007; Yang et al., 2013). NH_4_^+^ benefitted tea plant nitrogen metabolism and upregulated expression of ammonium-assimilation genes (Wang et al., 2021). However, knowledge of effects of NH_4_^+^ on the biosynthesis of tea components and molecular mechanisms involved has received little attention. To this end, Zhang et al. (2018) have established the limiting and sufficient nitrogen conditions for tea growth in a hydroponic system. This current work explored the effects of NH_4_^+^ deficiency and recovery by different ammonium concentrations on physiology and gene regulation in tea plants by the hydroponic method.

Advances in transcriptome analysis technologies have allowed thousands of genes related to nitrogen metabolism in tea plants to be identified. Evaluations of different N sources in tea plants (Liu et al., 2017; Yang et al., 2018; Wang et al., 2021) have found that NH_4_^+^ took precedence over NO_3_^–^ in assimilation. The work of Zhang et al. (2020) investigated the nitrogen uptake in tea roots and identified a group of modules related to low nitrogen treatment using weighted gene co-expression network analysis (WGCNA). Yang et al. (2020) focused on the relationship between nitrogen and amino acid metabolism. The current study explored the effects of NH_4_^+^ deficiency time and NH_4_^+^ concentration on gene expression in tea plants by hydroponics to reveal using RNA-seq coupled with a time-course experiment. Short- and long-term responses of tea plants to NH_4_^+^ deprivation and different concentrations of NH_4_^+^ resupply were assessed and a comprehensive and integrated dataset generated.

## Materials and methods

### Plant materials and experimental treatments

**FIGURE 1:**
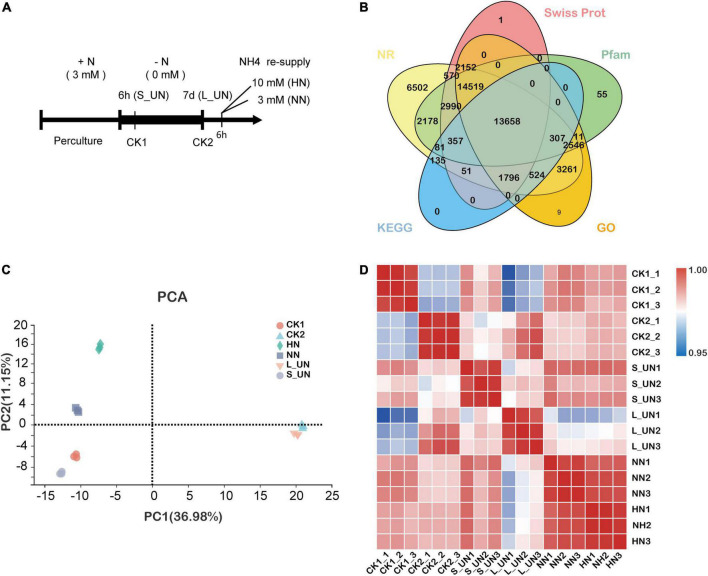
Experimental design and transcriptome relationships. **(A)** Schematic representation of the experimental design. After preculture, tea plants were transferred to N-deficient media (0 mM) for 6 h (S_UN) or 7 days (L_UN) or maintained in N-sufficient solution for 7 days (CK2). After treatment for 7 days, the N-starved plants were supplied with normal (NN) or high NH_4_^+^ (HN) media for up to 6 h. **(B)** Public gene expression databases. Each database is labeled with a colored oval. **(C)** Principal component analysis (PCA) of 18 RNA-Seq samples from “Longjing 43.” Each shape represents a different time point. **(D)** Pearson correlation coefficients of all samples (triplicates).

One-year-old “Longjing 43” tea seedlings were hydroponically cultivated in distilled water for 7 days and exposed to 1/4 strength nutrient solution for 1 week. The strength of the nutrient solution was thereafter increased to 1/2 (for around 2 weeks) and full (Zhang et al., 2018). The full nutrient solution contained the following nutrients: 1.5 mM (NH_4_)_2_SO_4_, 0.73 mM KNO_3_, 0.1 mM KH_2_PO_4_, 0.46 mM K_2_SO_4_, 0.41 mM MgSO_4_, 0.5 mM CaCl_2_, 0.046 mM H_3_BO_3_, 0.09 mM MnSO_4_, 0.0091 mM ZnSO_4_, 0.002 mM CuSO_4_, 0.0026 mM Na_2_MoO_4_, and 0.032 mM Fe-EDTA (Wang et al., 2022). After preculture for 1 month, tea seedlings with a similar appearance were chosen to be transferred to nutrient solution without N for 6 h (S_UN) or 7 days (L_UN) and controls were incubated at a normal NH_4_^+^ level (3 mM NH_4_^+^) for 6 h (CK1) or 7 days (CK2). After 1-week nitrogen deficiency, the nutrient solution was resupplied with 3 mM NH_4_^+^ (NN) or 10 mM NH_4_^+^ (high NH_4_^+^ level, HN) for 6 h (Figure 1A). The pH was adjusted to 5.0 ± 0.2 and solutions were refreshed every 4 days. All hydroponic solutions were continuously aerated using air pumps. Shoots with a bud and two leaves (three biological replicates for each treatment) were harvested, immediately snap-frozen in liquid nitrogen and stored at −80^°^C for analysis.

### Analysis of tea polyphenols and free amino acids

Each sample (0.15 g) was extracted with 25 mL of 50% methanol at 70^°^C for 20 min and centrifuged at 5,000 g for 10 min. The supernatants were collected for further analysis. The concentration of total polyphenols was determined using the Folin-Ciocalteu method. The content of total amino acids was determined by ninhydrin colorimetry according to Shao et al. (2008).

### RNA isolation, cDNA library construction and Illumina deep sequencing

Total RNA was extracted from the tissue using TRIzol reagent (Plant RNA Purification Reagent for plant tissue, Invitrogen), according to the manufacturer’s instructions and genomic DNA was removed using DNase I (TaKaRa). RNA quality was determined with a the 2100 Bioanalyzer (Agilent) and quantified by NanoDrop 2000. Only high-quality RNA samples (OD260/280 = 1.8∼2.2, OD260/230 ≥ 2.0, RIN ≥ 6.5, 28S:18S ≥ 1.0 > 1 μg) were used to construct the sequencing library.

An RNA-seq transcriptome library was prepared with TruSeq™ RNA sample preparation Kit from Illumina (San Diego, CA) using 1 μg of total RNA. Firstly, messenger RNA was isolated by polyA selection using oligo (dT) beads and then fragmented by fragmentation buffer. Double-stranded cDNA was synthesized using a SuperScript double-stranded cDNA synthesis kit (Invitrogen, CA) with random hexamer primers (Illumina). cDNA was subjected to end-repair, phosphorylation and “A” base addition according to the Illumina library construction protocol. Libraries were size-selected for cDNA target fragments of 300 bp on 2% low range ultra agarose followed by PCR amplification using Phusion DNA polymerase (NEB) for 15 PCR cycles. After quantifying by TBS380, the paired-end RNA-seq sequencing library was sequenced using the Illumina HiSeq XTen/NovaSeq 6000 sequencer (2 × 150 bp read length).

### Mapping the RNA-seq reads

The raw paired-end reads were trimmed and quality controlled by using the SeqPrep^1^ and Sickle software^2^ with the default parameters. Clean reads were aligned to the tea plant reference genome^3^ with orientation mode using HISAT2 software^4^ (Kim et al., 2015). Mapped reads of each sample were assembled using StringTie software^5^ in a reference-based approach (Pertea et al., 2015).

### Differential expression analysis and functional enrichment analysis

In order to identify differentially expressed genes (DEGs) between two different samples, the expression level of each transcript was calculated according to the fragments per kilobase per million reads (FPKM) method. RSEM^6^ (Li and Dewey, 2011) was used to quantify gene abundance. differential expression analysis was performed using the DESeq2 tool (Love et al., 2014) with FDR<0.05, such that DEGs with |log2FC| ≥ 1 and FDR < 0.05 were considered to have a significantly different expression. In addition, KOBAS^7^ (Xie et al., 2011) and Goatools^8^ was used for functional enrichment analyses, including gene ontology (GO) and Kyoto encyclopedia for genes and genomes (KEGG) to identify significantly overrepresented GO terms and metabolic pathways according to the Bonferroni-corrected *p*-value ≤ 0.05 compared with the whole-transcriptome background. WGCNA was performed as described by Langfelder and Horvath (Langfelder and Horvath, 2008) and data of all DEGs among the different treatments analyzed. The gene co-expression network was visualized using Cytoscape software (version 3.5.1).

### RNA-seq data validation using quantitative real-time PCR

Total RNA was used to synthesize the first strand cDNA using PrimeScript™ RT reagent Kit with gDNA Eraser Perfect Real Time (Takara, Japan). RT-qPCR was conducted with SYBR Green reagents (Takara, Japan) using an ABI StepOne Plus real-time PCR machine (Applied Biosystems). The PCR program was as follows: 94^°^C for 5 min, 40 cycles of 30 s at 94^°^C, 30 s at 60^°^C and a final melting curve at 65−95^°^C. GADPH was used as internal reference gene to quantify cDNA. The threshold cycle (Ct) values of the PCR were averaged and the relative transcript levels were quantified using the 2^–△△^^Ct^ method. All primers used for the RT-qPCR are listed in Supplementary Table 1.

### Statistical analysis

One-way ANOVA (SPSS 25.0, SPSS Inc., Chicago, IL, United States) and Bonferroni tests (*p* < 0.05) was used to detect significant differences. All data are presented as means ± *SD*.

## Results and discussion

### Experimental design and RNA sequence analysis

Six time points and three biological replicates per condition were used to construct RNA-seq libraries for analysis of the effects of NH_4_^+^ status on gene expression in tea shoots. Quality check were performed to remove reads containing adapter, ploy-N and with low quality from the raw data, leaving an average number of clean reads per library above 6.13 Gb and a total of 130.59 Gb of clean reads. Approximately 91.04–92.25% of the clean reads were successfully mapped to the *Camellia sinensis* genome and 84.50–85.01% of the clean reads matched unique genomic locations. Each of the transcriptomes had 44.40–45.41% GC content. The values of Q30 were between 93.52 and 94.42% (Supplementary Table 2). These results suggested that the RNA sequencing data used in the present study were highly reliable for *de novo* assembly and expression analysis. The BLAST tool was used to assess sequence similarity against five public databases: GO, KEGG, NR, Swiss Prot, and Pfam to assign potential functions to the assembled unigenes (Figure 1B).

The results of PCA analysis are shown in Figure 1C. PC1 accounted for 36.98% of the total variance, with clearly separated samples according to the duration of nitrogen deficiency. PC2 accounted for 11.15% of the total variance with samples separated according to different NH_4_^+^ concentrations, such that the samples with a high concentration showed higher PC2 values (Figure 1C). Three biological replicates of each sample were clustered together, indicating high data reproducibility. Overall, the PCA analysis results showed that both the time and concentration of nitrogen supply could affect the gene expression patterns of tea plants. In addition, development status also affected gene expression. Pearson correlation coefficients of the FPKM distribution among biological replicates of all treatments ranged from 0.958 to 0.997 (Figure 1D), indicating a high reproducibility of the sequencing data.

### Analysis of responses to short- and long-term NH_4_^+^ starvation

**FIGURE 2:**
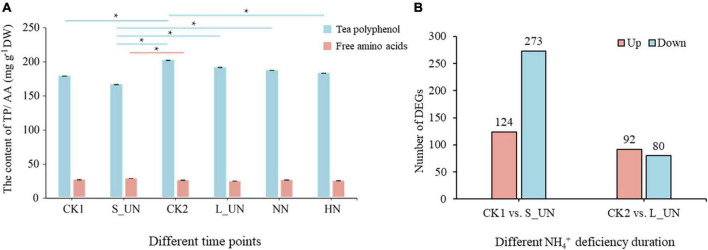
An overview of different periods of NH_4_^+^ treatments. **(A)** The content of tea polyphenols and free amino acids in tea plants at different time points. **(B)** Differentially expressed genes between different NH_4_^+^ deficiency durations (S_UN vs. CK1 and L_UN vs.CK2). * indicates a significant difference (*p* < 0.05).

Tea polyphenols and free amino acids are the two most important components of tea plants and are affected by nitrogen supply. The content of tea polyphenols (TP) increased with time, being higher after 7 days. TP content was higher after long-term nitrogen deficiency (L_UN) than after short-term nitrogen deficiency (S_UN) (Figure 2A). Development status had great influence on the content of tea polyphenols and free amino acids. DEGs involved in plant growth were eliminated to enhance the accuracy of experimental results. There were 397 DEGs in short-term nitrogen starvation plants compared with CK1, and most of them (273 genes) were downregulated (Figure 2B). After long-term nitrogen starvation, 172 DEGs were identified, including 92 upregulated and 80 downregulated. Thus, tea plants have a more obvious response to short-term NH_4_^+^ deficiency. The number of DEGs after long-term NH_4_^+^ deficiency may have been fewer because gene expression tended to stabilize after a long period of NH_4_^+^ deprivation.

**FIGURE 3:**
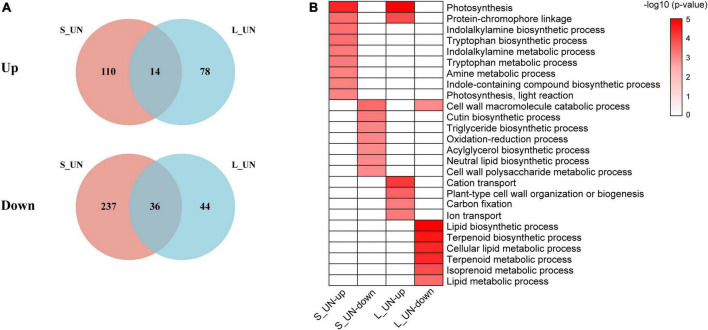
Venn diagram and functional analysis of DEGs of different time periods of NH_4_^+^ deficiency. **(A)** Venn diagram of the number of upregulated and downregulated DEGs between short-term NH_4_^+^ deficiency and long-term NH_4_^+^ deficiency. **(B)** Significantly enriched GO terms (biological process) of specific DEGs between different NH_4_^+^ deficiency durations (Bonferroni’s test, *p* < 0.05).

A total of 14 upregulated and 36 downregulated genes were common to short- and long-term nitrogen deficiency groups (Figure 3A), indicating that duration of nitrogen deficiency affected the expression of different genes. According to GO enrichment analysis, upregulated genes were involved in photosynthesis and protein-chromophore linkage in both long-term and short-term nitrogen deficiency (Figure 3B), suggesting a link between nitrogen deficiency and photosynthesis. Photosynthesis genes, including those encoding photosystem P700 chlorophyll a apoprotein A2 (psaB) (TEA002548), acetyl-CoA carboxylase transferase (chloroplast) (TEA027086) and ribulose bisphosphate carboxylases (Rubiscos) (TEA020293) were significantly upregulated by N deficiency (Supplementary Table 3). When nitrogen is scarce, photosynthesis becomes limited and the expression of genes related to the phenylpropanoid pathway (*PAL, CHS*, and *CHI*) are known to be up-regulated (Dong et al., 2019). Downregulated genes were enriched in the GO term, cell wall macromolecule catabolic (Figure 3B). Cell wall remodeling represents an important characteristic of plant growth and differentiation (Krouk et al., 2010). The results showed that nitrogen deficiency resulted in slow cell wall remodeling. Biological process in GO terms related to photosynthesis, plant hormone and amino acid metabolism was greatly affected during the early stage of the nitrogen deficiency response. The enhancement of these metabolic pathways may result from the lack of nitrogen disrupting the carbon/nitrogen balance. By contrast, regulations of genes involved in ion transport, lipid and terpenoid metabolism were affected by a long period of nitrogen deficiency. Long-term nitrogen deficiency not only affected carbon fixation in photosynthesis (Makino, 2011), but also inhibited the TCA cycle and fatty acid synthesis (Bouché and Fromm, 2004). Previous studies on Arabidopsis thaliana have shown that PII proteins that regulate nitrogen metabolism was also involved in regulating fatty acid synthesis and metabolism through interaction with the biotin carboxyl carrier protein BCCP (Baud et al., 2010). These results indicate the importance of nitrogen in the growth and metabolism of tea plants.

### Analysis of normal and high NH_4_^+^ concentration recovery

**FIGURE 4:**
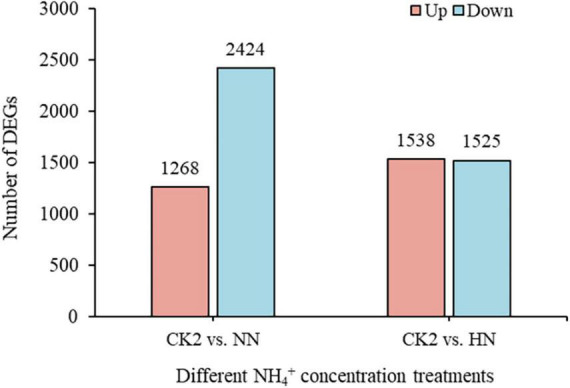
The number of DEGs under different NH_4_^+^ resupply concentrations (HH vs. CK2 and NN vs. CK2).

Following nitrogen deficiency, different gene expression patterns with different NH_4_^+^ resupply concentrations were found. A total of 3,692 DEGs were found, including 1,268 upregulated and 2,424 downregulated at normal resupply concentration of NH_4_^+^ (Figure 4). With high NH_4_^+^ resupply concentration, there were 3,063 DEGs, with nearly equal numbers of upregulated and downregulated genes (Figure 4). NH_4_^+^ resupply enhanced the changes in gene expression in tea plants, reflecting the rapid and obvious response of tea plants to NH_4_^+^.

**FIGURE 5:**
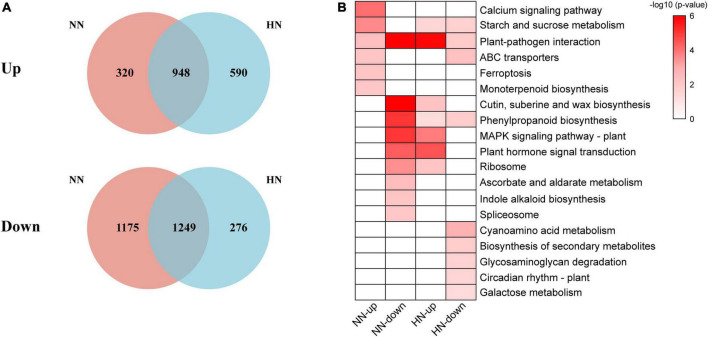
Venn diagram and functional analysis of DEGs under different NH_4_^+^ resupply concentrations. **(A)** Venn diagram of DEGs (upregulated and downregulated genes) under different NH_4_^+^ resupply concentrations. **(B)** Significantly enriched KEGG pathways of specific DEGs under different NH_4_^+^ resupply concentrations (Bonferroni’s test, *p* < 0.05).

The influence of different NH_4_^+^ concentrations on the gene expression patterns of tea plants may be seen in the Venn diagram in Figure 5A. At normal NH_4_^+^ concentration, there were 1,428 unique genes (320 upregulated and 1,175 downregulated) while there were 799 unique genes (590 upregulated and 276 downregulated) with high NH_4_^+^ concentration resupply. Thus, normal and high NH_4_^+^ concentrations may have different gene expression models for tea plants. Regardless of NH_4_^+^ concentration, starch and sucrose metabolism, phenylpropanoid biosynthesis and plant-pathogen interaction were the main response pathways after NH_4_^+^ resupply (Figure 5B), suggesting an impact on metabolism of nitrogen resupply.

Interestingly, the results showed that the downregulated genes with normal NH_4_^+^ resupply were similar to those upregulated genes with high NH_4_^+^ resupply (Figure 5B). In addition, DEGs were related to plant-pathogen interaction, MAPK signaling, and plant hormone signaling which may account for considerable environmental adaptability. It had also been previously found that the ammonium-specific response is related to biological stress and plant defense (Patterson et al., 2010). High NH_4_^+^ content can protect the plants from pathogens, increase the cross-tolerance to other forms of abiotic stresses and improve the quality of crops (Marino and Moran, 2019). The genes involved in the plant-pathogen interaction, including leucine-rich repeat protein, LRR receptor-like serine/threonine-protein kinase, cyclic nucleotide-gated ion channel (CNGC), disease resistance protein, ethylene-responsive transcription factor, and transcription factor WRKY were significantly highly expressed with NH_4_^+^ resupply. These results have rarely been reported in tea plants. In summary, our results revealed that the signaling role of the NH_4_^+^ molecule.

### Identification of gene co-expression modules using weighted gene co-expression network analysis

**FIGURE 6:**
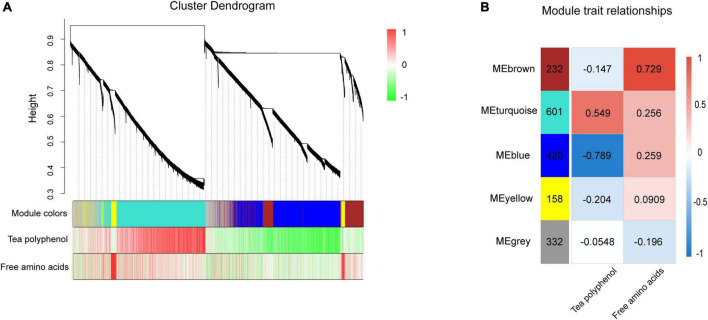
WGCNA of genes in tea leaves under different NH_4_^+^ treatments. **(A)** Gene cluster dendrogram and relationships between the contents of TP, AA and modules. According to the expression trend of transcripts, transcripts were divided into modules, where branches represent a gene, and a color represents a module. **(B)** Heatmap of correlation between module and trait. The vertical axis represents different modules, the left column of numbers represents the number of genes in the module and each set of data on the right represents the correlation coefficient.

WGCNA was used to comprehensively understand the gene expression patterns related to NH_4_^+^ treatments in tea plants. After removing the genes with low variability, five modules were identified, as shown in Figure 6. After generating a summary profile for each module, the turquoise module, containing 601 genes, was highly correlated with tea polyphenols, while the brown module, containing 232 genes, was correlated with free amino acids (Figure 6). Tea polyphenols and free amino acids are the two most important components of tea plants. Fewer genes were associated with free amino acids than with tea polyphenols. These results suggested that the effect of NH_4_^+^ on tea polyphenols was more significant compared with that of free amino acids, consistent with previous research (Liu et al., 2017). The top 30 associated genes according to the weighting values were selected from the turquoise and brown modules to construct a network (Supplementary Table 4). Hub genes are genes with the most connections in the network, as shown by their degree.

**FIGURE 7:**
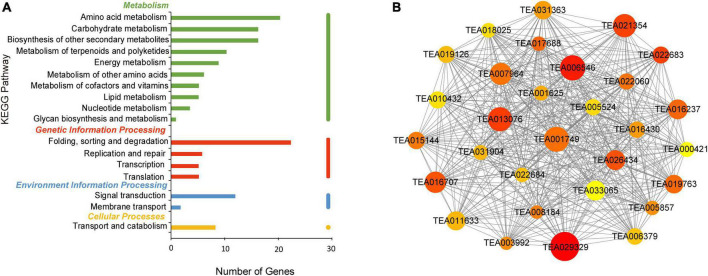
KEGG pathway analysis and network diagram associated with the turquoise module. **(A)** KEGG pathway annotation of genes in the turquoise module. The genes with higher weight are shown in red, and the size of the node represents the number of genes associated with the node. **(B)** Gene networks of hub genes of the turquoise module.

In the turquoise module, 20 genes were enriched in amino acid metabolism. Both carbohydrate metabolism and biosynthesis of other secondary metabolites contained 17 genes according to KEGG analysis, as shown in Figure 7A. Photosynthesis represents the basis of plant metabolism and energy source and influences tea polyphenols formation. Carbonic anhydrase (*CA*) (TEA013076) is related to carbon utilization and was one of the hub genes in photosynthesis. Phytochrome B (TEA031363) was an important gene. Tea polyphenols assist the resistance of plants to damage by external factors, such as UV-B, low temperature, and drought (Zoratti et al., 2014). Genes encoding for the REF/SRPP-like protein (TEA001749), E3 ubiquitin ligase (TEA022683) and carboxylesterase (CXE) (TEA001625) were involved in plant stress resistance (Tong et al., 2017; Cao et al., 2019; Choi et al., 2021). Glutathione S-transferase (*GST*) (TEA006546) was also a hub gene and involved in normal metabolism of plant secondary products, such as flavonoids and cinnamic acid (Marrs, 1996). Other hub genes identified included glutathione synthetase (*GS*) (TEA017688) and altered xyloglucan (*AXY*) (TEA016707) (Figure 7B). These results suggest that nitrogen deficiency may be an abiotic stress that produces reactive oxygen species (ROS). The accumulation of tea polyphenols with high antioxidant activity may contribute to ROS scavenging (Kovacik et al., 2014). Although these genes are not directly involved in the formation of tea polyphenols, they are related to tea plant growth and metabolism, *via* processes such as photosynthesis, plant stress and carbon and nitrogen balance (Margaria et al., 2014; Wang et al., 2021).

**FIGURE 8:**
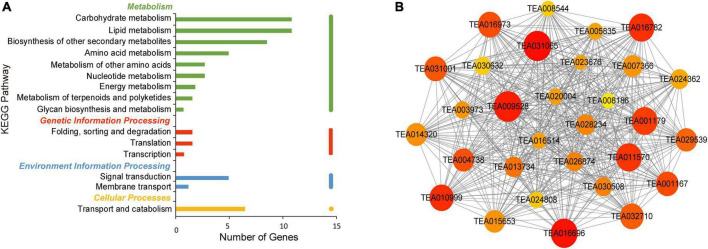
KEGG pathway analysis and network diagram associated with the brown module. **(A)** KEGG pathway annotation of genes in the brown module. The genes with higher weight are shown in red, and the size of the node represents the number of genes associated with the node. **(B)** Gene networks of hub genes of the brown module.

In the brown module, enriched genes were involved in carbohydrate and lipid metabolism and biosynthesis of other secondary metabolites (Figure 8A). The co-expression network is shown in Figure 8B. Cytochrome P450 86A22 (TEA020004) was found to be a hub gene and is a key fatty acid acyl coenzyme essential for the synthesis of esters in petunia (Han et al., 2010). Hub genes included 3-ketoacyl-CoA synthase (*KCS*) (TEA005835), a rate-limiting enzyme involved in the elongation of very long fatty acid chains (Yang et al., 2021). In rice, the germin-like protein regulates plant height and disease resistance (Banerjee and Maiti, 2010). GDSL esterases/lipases (TEA032710) have also been identified as hub genes and are mainly involved in the regulation of plant development, synthesis of secondary metabolites and the defense response (Chepyshko et al., 2012). Moreover, pathogenesis related protein and germin-like protein are thought to be involved in the response to abiotic stress in plants (Kapoor et al., 2019). In addition, laccase (*LAC*) (TEA004738) is a hub gene related to lignin synthesis, disease resistance and pigment synthesis (Li and Steffens, 2002; Ranocha et al., 2002; Pourcel et al., 2005). These results demonstrated the complex network system connecting synthesis of free amino acids with many pathways in tea plants.

### Validation of the differentially expressed genes by RT-qPCR

**FIGURE 9:**
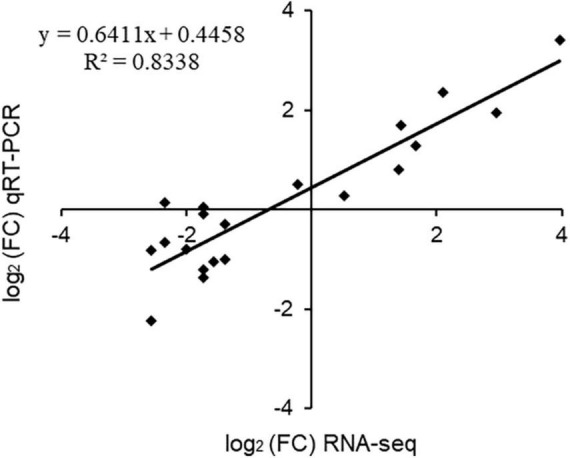
Scatterplot of RNA-seq transcript abundance fold change values in RNA-Seq (on the *x* axis) against RT-PCR expression values (on *y* axis) for the 20 selected genes by RT-qPCR. The GADPH gene (XM_002263109) was used as the reference gene.

Twenty genes from different expression profiles were chosen for RT-qPCR analysis to validate the quality of the RNA-Seq data. Genes belonged to different metabolic pathways, including photosynthesis (photosystem II, chlorophyllase 1), polyphenol synthesis [leucoanthocyanidin reductase (*LAR*), phalcone synthase (*CHS*), flavone synthase II], amino acid synthesis (terpene synthase), anti-resistant genes (pathogenesis-related protein, disease resistance protein) and some transporters and transcription factors. The expression value of RT-qPCR was positively correlated with log_2_ fold change of RNA sequencing results (*R*^2^ = 0.8338), indicating the reliability of our transcriptional data (Figure 9). The expression levels of sugar transporter (TEA027183), disease resistance protein (TEA023317) and flavone synthase (TEA012257) genes were increased under high concentration of NH_4_^+^. High NH_4_^+^ concentrations, therefore, had a stress effect on tea plants. In summary, tea plants prefer NH_4_^+^ within a defined concentration range (Ruan et al., 2007).

## Data availability statement

The data presented in this study are deposited in the NCBI repository, accession number PRJNA844353 (https://www.ncbi.nlm.nih.gov/bioproject/PRJNA844353).

## Author contributions

YW and J-XO: data curation. YW, Y-MX, and D-MF: formal analysis. X-CW and X-QZ: funding acquisition, writing—review, and editing. X-CW: project administration. YW: resources and writing—original draft. All authors have read and agreed to the published version of the manuscript.

## Footnotes

1
https://github.com/jstjohn/SeqPrep

2
https://github.com/najoshi/sickle

3
http://tpia.teaplant.org/index.html

4
http://ccb.jhu.edu/software/hisat2/index.shtml

5
https://ccb.jhu.edu/software/stringtie/

6
http://deweylab.biostat.wisc.edu/rsem/

7
http://kobas.cbi.pku.edu.cn/home.do

8
https://github.com/tanghaibao/Goatools

## Supplementary material

Supplementary Table 4 Top 30 genes in WGCNA modules.
